# Neuronal transmission of timing precision: dependence on intrinsic and synaptic properties

**DOI:** 10.1186/1471-2202-13-S1-P177

**Published:** 2012-07-16

**Authors:** Heather A Brooks, Alla Borisyuk

**Affiliations:** 1 Department of Mathematics, University of Utah, Salt Lake City, UT 84112, USA

## 

Precision of spike timing and its role in information processing is one of the key themes in systems neuroscience. As signals travel through nervous systems, the precision and reliability of the timing information is altered. Interestingly, depending on the particular system, the timing precision can be improved or reduced through propagation. For example, at the transmission from auditory nerve to brainstem the emergence of latency precision and jitter reduction is observed [1]. On the other hand, phase locking to stimulus deteriorates across levels of auditory system from very good phase locking at early stages of processing in the brainstem to weaker phase locking in the midbrain (inferior colliculus) to an even weaker one in the auditory cortex [2]. This raises the issue of which properties allow for the improvement or deterioration of the timing information.

Motivated by the processing of timing information in the auditory system, we examine the cellular and synaptic properties that affect improving or decreasing the precision of spike timing through synaptic transmission.

We show through combination of analysis and numerics in minimal neuronal models (integrate-and-fire and Morris-Lecar) that both improvement and deterioration of spike-time precision through transmission is possible, depending on the input times distribution, number of inputs and synaptic strength.

Further, in a conductance-based model we include more nuanced intrinsic and synaptic properties, in particular, currents that switch the neuron from integrator to coincidence detector (Prescott et al., 2006; Svirskis et al., 2004). We characterize response properties that deviate from the performance of an “ideal” integrator and coincidence detector in a manner dependent on details of the intrinsic ionic currents.
